# Supplementary material for: A Comparison of Vaping Behavior, Perceptions, and Dependence among Individuals Who Vape Nicotine, Cannabis, or Both
Source: Int J Environ Res Public Health. 2022 Aug 20;19(16):10392. doi: 10.3390/ijerph191610392 (PMC9408799; doi:10.3390/ijerph191610392)
Supplement: Supplementary file 1 [file ijerph-19-10392-s001.zip › ijerph-1864357-supplementary.pdf]

File S1. The full online assessment battery administered during the second wave of the survey.

Note: Validity and attention check items are italicized.

Section I: Eligibility

1. Have you read the Summary of Explanation of Research?
    a. Yes
    b. No
2. Have vaped or used an electronic cigarette in the past 30 days?
    a. Yes
    b. No
3. Am 18 years of age or older?
    a. Yes
    b. No
4. Can read and write in English
    a. Yes
    b. No
5. Am not a member of the European Union General Data Protection Regulation (GDPR)?
    a. Yes
    b. No

Section II: Product Screening

1. How old are you?
2. Have you ever used cannabis (including: marijuana, cannabis, THC, or CBD)?
    a. Yes
    b. No
3. Have you used a vape pen or e-cigarette that contains nicotine in the past 30 days?
    a. Yes
    b. No
4. Have you used a vape pen or e-cigarette that contains cannabis, marijuana, THC, or CBD in the past 30 days?
    a. Yes
    b. No

Section III: Demographics

1. Do you consider yourself Hispanic/Latino?
    a. Yes
    b. No
2. What is your Hispanic/Latino origin or ancestry?
    a. 1, Puerto Rican
    b. 2, Dominican Republic
    c. 3, Mexican/Mexicano
    d. 4, Mexican American, Chicano
    e. 5, Cuban, Cuban American

    f. 6, Central or South American
    g. 7, Other Latin American, Other Hispanic
3. Which race best describes you?
    a. 1, Caucasian/white
    b. 2, African American/black
    c. 3, Asian
    d. 4, Native Hawaiian or Pacific Island
    e. 6, American Indian/Alaskan native
    f. 5, Other
4. What is the highest level of school you have completed or the highest degree you have received?
    a. 1, Less than 9th grade
    b. 2, 9th grade
    c. 3, 10th grade
    d. 4, 11th grade
    e. 5, 12th grade/no diploma
    f. 6, High school graduate
    g. 7, GED or equivalent
    h. 8, Some college/no degree
    i. 9, Associate degree
    j. 10, Bachelor's degree
    k. 11, Master's degree
    l. 12, Professional Degree (ex: MD, DDS, DVM, JD)
    m. 13, Doctoral degree (ex: PhD, EdD)
5. Are you currently enrolled in a degree program?
    a. Yes
    b. No
6. Is it a technical or vocational program, a 2-year degree program, or a 4-year degree program, or a graduate or professional program?
    a. 1, Technical/vocational program
    b. 2, 2-year degree program
    c. 3, 4-year degree program
    d. 4, Graduate/professional program
7. Which of these best describes your current employment status?
    a. 1, WORKING NOW FULL-TIME
    b. 2, WORKING NOW PART-TIME
    c. 3, ONLY TEMPORARILY LAID OFF, SICK LEAVE OR MATERNITY LEAVE
    d. 4, LOOKING FOR WORK, UNEMPLOYED
    e. 5, RETIRED
    f. 6, NON-WORKING DISABLED, PERMANENTLY OR TEMPORARILY
    g. 7, KEEPING HOUSE
    h. 8, MILITARY
    i. 9, NON-WORKING STUDENT
    j. 88, DON'T KNOW

8. Which of these best describes your relationship status?
    a. 1, Married
    b. 2, Widowed
    c. 3, Divorced
    d. 4, Separated
    e. 5, Never Married
    f. 6, Living with a partner
    g. 7, Member of an unmarried couple (not living together)
9. What is your gender identity?
    a. 1, Male (male assigned at birth, male-spectrum identity)
    b. 2, Female (female assigned at birth, female-spectrum identity)
    c. 3, Transmale/transman/Female to male (female assigned at birth, male-spectrum identity)
    d. 4, Transfemale/transwoman/Male to female (male assigned at birth, female-spectrum identity)
    e. 5, Other identity
    f. 6, Choose not to disclose
10. What is your sexual orientation?
    a. 1, Bisexual (a person who is sexually attracted to both men and women)
    b. 2, Gay (a homosexual man)
    c. 3, Lesbian (a homosexual woman)
    d. 4, Straight/Heterosexual (a person sexually attracted to people of the opposite sex
    e. 5, Other
    f. 6, Choose not to disclose
11. Was your family's total combined income from all sources before taxes last year less than $20,000 or $20,000 or more?
    a. Less than $20,000
        i. Which of the following income categories best describes your total household income last year?
            1. Less than $1,000
            2. $1,000 to $1,999
            3. $2,000 to $2,999
            4. $3,000 to $3,999
            5. $4,000 to $4,999
            6. $5,000 to $5,999
            7. $6,000 to $6,999
            8. $7,000 to $7,999
            9. $8,000 to $8,999
            10. $9,000 to $9,999
            11. $10,000 to $10,999
            12. $11,000 to $11,999
            13. $12,000 to $12,999
            14. $13,000 to $13,999

15. $14,000 to $14,999
16. $15,000 to $15,999
17. $16,000 to $16,999
18. $17,000 to $17,999
19. $18,000 to $18,999
20. $19,000 to $19,999
21. Don't know
22. Refused

b. 2, More than $20,000

1. $20,000 to $20,999
2. $21,000 to $21,999
3. $22,000 to $22,999
4. $23,000 to $23,999
5. $24,000 to $24,999
6. $25,000 to $25,999
7. $26,000 to $26,999
8. $27,000 to $27,999
9. $28,000 to $28,999
10. $29,000 to $29,999
11. $30,000 to $30,999
12. $31,000 to $31,999
13. $32,000 to $32,999
14. $33,000 to $33,999
15. $34,000 to $34,999
16. $35,000 to $35,999
17. $36,000 to $36,999
18. $37,000 to $37,999
19. $38,000 to $38,999
20. $39,000 to $39,999
21. $40,000 to $44,999
22. $45,000 to $49,999
23. $50,000 to $54,999
24. $55,000 to $59,999
25. $60,000 to $64,999
26. $65,000 to $69,999
27. $70,000 to $74,999
28. $75,000 to $79,999
29. $80,000 to $84,999
30. $85,000 to $89,999
31. $90,000 to $94,999
32. $95,000 to $99,999
33. $100,000 and over
34. Don't know

35. Refused
c. Don't know

12. I am paid bi-weekly by leprechauns
a. Disagree
b. Slightly Disagree
c. Agree
d. Strongly Agree

Section IV: Tobacco History

1. Have you smoked at least 100 cigarettes in your entire life? NOTE: 5 packs = 100 cigarettes
   a. 1, Yes
   b. 2, No
   c. 7, Don't know / Not sure
   d. 9, Refused
2. Do you now smoke cigarettes every day, some days, or not at all?
   a. 1, Every day
   b. Some days
   c. Not at all
   d. Don't know / Not sure
   e. Refused
3. How long has it been since you last smoked cigarettes regularly?
   a. 01, Within the past month (less than 1 month ago)
   b. Within the past 3 months (1 month but less than 3 months ago)
   c. Within the past 6 months (3 months but less than 6 months ago)
   d. Within the past year (6 months but less than 1 year ago)
   e. Within the past 5 years (1 year but less than 5 years ago)
   f. Within the past 10 years (5 years but less than 10 years ago)
   g. 10 years or more
   h. Never smoked regularly
   i. Don't know / Not sure
   j. Refused
4. Think of the time during your life when you smoked the most. During that time, how many cigarettes did you smoke each day?
5. When you wake up in the morning, please rate your urge to smoke with 1 being the lowest and 10 being the highest.
   a. 1
   b. 2
   c. 3
   d. 4
   e. 5
   f. 6
   g. 7

    h. 8
    i. 9
    j. 10
6. Do you usually smoke menthol or non-menthol?
    a. Menthol
    b. Non-menthol
7. How old were when you first started smoking cigarettes fairly regularly?
8. Think about a time in your life when your smoking was at its heaviest. If you are smoking less than at that time, what is causing you to smoke less now?
    a. I have reduced to improve my health
    b. I have reduced in preparation for quitting
    c. Can't smoke in my home
    d. Can't smoke at work
    e. I have reduced to save money
    f. I have not reduced (still smoking at my heaviest)
    g. Other
9. Please describe why.

For the next few questions, think about quit attempts that you have EVER MADE.

10. Have you EVER made a serious attempt to stop smoking because you were TRYING to quit -- even if you stopped for less than a day?
    a. Yes
    b. No
11. How many times have you ever made a serious attempt to stop smoking because you were TRYING to quit -- even if you stopped for less than a day?
12. Of all the quit attempts you have made, how long did your longest quit attempt last? Was it days, months, or years?
    a. Days
    b. Months
    c. Years
13. How many days did your longest quit attempt last?
14. How many months did your longest quit attempt last?
15. How many years did your longest quit attempt last?
16. On your longest previous quit attempt, did you gain any weight?
    a. Yes
    b. No
17. How much weight did you gain on this quit attempt?
18. During any of your past quit attempts, did you use any of the following products: nicotine patch, nicotine gum/lozenge, nicotine nasal spray, nicotine inhaler, electronic cigarette, Chantix or Varenicline, Zyban, Bupropion or Wellbutrin or any other product?
    a. Yes
    b. No
19. Please tell us which products you used (mark all that apply):
        a. A nicotine patch

b. A nicotine gum or nicotine lozenge
c. A nicotine nasal spray
d. A nicotine inhaler
e. An electronic cigarette
f. A prescription pill called Chantix or Varenicline
g. A prescription pill called Zyban, Bupropion, or Wellbutrin
h. Another prescription pill
i. Another cessation product
j. Please specify the other type of prescription pill
k. Please specify the other cessation product

For the next few questions: think about ONLY quit attempts you have made DURING THE PAST 12 MONTHS.

20. DURING THE PAST 12 MONTHS, have you made a serious attempt to stop smoking because you were TRYING to quit -- even if you stopped for less than a day?
    a. Yes
    b. No

21. How many times DURING THE PAST 12 MONTHS have you made a serious attempt to stop smoking because you were TRYING to quit -- even if you stopped for less than a day?
22. Currently, do you have any symptoms or a disease that you believe is caused or made worse by your tobacco use?
    a. Yes
    b. No
23. If yes, please describe
24. Does smoking help you cope with stress?
    a. Yes
    b. No

Section V: Nicotine Dependence

1. How soon after you wake do you smoke your first cigarette of the day?
    a. Within 5 minutes
    b. 6-30 minutes
    c. 31- 60 minutes
    d. After 60 minutes
2. Do you find it difficult to refrain from smoking in places where it is forbidden?
    a. Yes
    b. No
3. Which cigarette would you hate to give up most?
    a. First one in the morning
    b. Any other
4. How many cigarettes per day do you usually smoke?
    a. 10 or less
    b. 11-20

c. 21-30
d. 31 or more

5. Do you smoke more frequently during the first hours after awakening than during the rest of the day?
   a. Yes
   b. No
6. Do you smoke even if you are so ill that you are in bed most of the day?
   a. Yes
   b. No
7. Do you sometimes awaken at night to have a cigarette?
   a. Yes
   b. No
8. If yes, how many nights per week do you typically awaken to smoke?
9. Over the past week, how strong have your urges to smoke been?
   a. No urges
   b. Slight
   c. Moderate
   d. Strong
   e. Very strong
   f. Extremely strong
10. How many cigarettes per day do you usually smoke?
11. On days that you can smoke freely, how soon after you wake up do you smoke your first cigarette of the day?
12. How much of the time have you felt the urge to smoke in the past 24 hours?
    a. Not at all
    b. A little of the time
    c. Some of the time
    d. A lot of the time
    e. Almost all of the time
    f. All of the time
13. How strong have the urges been in the past 24 hours?
    a. No urges
    b. Slight
    c. Moderate
    d. Strong
    e. Very strong
    f. Extremely strong
14. Have you ever tried to quit but couldn't?
    a. Yes
    b. No
15. Do you smoke now because it is really hard to quit?
    a. Yes
    b. No
16. Have you ever felt like you were addicted to tobacco?

a. Yes
b. No

17. Do you ever have strong cravings to smoke?
    a. Yes
    b. No
18. Have you ever felt like you really needed a cigarette?
    a. Yes
    b. No
19. Is it hard to keep from smoking in places where you are not supposed to?
    a. Yes
    b. No
20. ...did you find it hard to concentrate because you couldn't smoke?
    a. Yes
    b. No
21. ...did you feel more irritable because you couldn't smoke?
    a. Yes
    b. No
22. ...did you feel a strong need or urge to smoke?
    a. Yes
    b. No
23. ...did you feel nervous, restless or anxious because you couldn't smoke?
    a. Yes
    b. No
24. You missed at least one question above, please complete all questions on this page prior to moving forward with the next survey. If you cannot answer the questions you left blank because either you are uncomfortable with the question or you don't know the answer then please check this box: I do not wish to answer question(s) that are left blank

Section VI: Nicotine E-Cigarette Use History

Instructions: The following questions are about your use of vaporized nicotine e-liquid. Nicotine e-liquid can be used in pod based devices, electronic cigarettes, vapes, or other products that provide vaporized nicotine. For this questionnaire, we are NOT asking about other ways that you might use nicotine (e.g., combustibles, chew, snuff). We are also NOT asking about your use of vapes that contain other types of e-liquid. (e.g., THC, CBD, flavorants only).

1. We refer to your electronic nicotine vaping device as an electronic cigarette or e-cig in this survey. What do you call your device?
2. At what age did you first try an electronic cigarette?
3. At what age did you first begin to use an electronic cigarette regularly?
4. How long have you been using an electronic cigarette? Days, months, or years?
    a. Days
    b. Months

c. Years
5. How many days?
6. How many months?
7. How many years?
8. What type of nicotine product were you using? (check all that apply)
   a. Cigarettes
   b. Snus/snuff/chew
   c. Cigars
   d. Hookah
   e. Other
9. On how many days of the past 30 have you used an electronic cigarette?
10. How often do you "stealth vape" by using your e-cigarette discreetly in places or situations where it may not be allowed or socially acceptable?
    a. Never
    b. Rarely
    c. A few times a month
    d. A few times a week
    e. Daily
11. What type of e-cig do you use most often? Please select the type that most closely resembles your device.
    a. Type 1
    b. Type 2
    c. Type 3
    d. Type 4
12. Where do you purchase the e-liquid that you use in your device?
13. If you purchase your products somewhere other than listed above, please write in:
14. What nicotine concentration do you use most often? Please enter in mg/ml.
15. What flavor e-liquid do you typically use?
    a. None
    b. Tobacco
    c. Menthol
    d. Mint
    e. Other
16. What flavor do you typically use?
17. Do you use more than one flavor?
18. What other flavors do you use?
19. Do you ever feel as though the e-liquids you purchase have been unsafe?
    a. Yes
    b. No
20. Do you have a method of verifying that the e-liquid you purchased is the product you intended to receive?
    a. Holding e-liquid up to light
    b. Looking for bubble in e-liquid
    c. Looking at packaging for damage

   d. Smelling the e-liquid
   e. Checking the viscosity
   f. Other
21. Please tell us the other ways you verify the e-liquid.
22. Have you had any concerns for your health due to your e-cigarette use?
23. Have you previously used your e-cig device for vaping liquids containing products other than nicotine?
24. What other liquids?
25. Do you use an e-cigarette to replace the use of regular (combustible) cigarettes?
26. For the next few questions, think about quit attempts that you have EVER MADE.
27. Have you EVER made a serious attempt to stop vaping because you were TRYING to quit -- even if you stopped for less than a day?
   a. Yes
   b. No
28. How many times have you ever made a serious attempt to stop vaping because you were TRYING to quit -- even if you stopped for less than a day?
29. Of all the quit attempts you have made, how long did your longest quit attempt last? Was it days, months, or years?
   a. Days
   b. Months
   c. Years
30. How many days did your longest quit attempt last?
31. How many months did your longest quit attempt last?
32. How many years did your longest quit attempt last?
33. On your longest previous quit attempt, did you gain any weight?
   a. Yes
   b. No
34. How much weight did you gain on this quit attempt?
35. How important is losing weight or maintaining your current weight compared with other personal health concerns? (if 1 is not at all and 10 is extremely important)
   a. 1- Not at all
   b. 2
   c. 3
   d. 4
   e. 5
   f. 6
   g. 7
   h. 8
   i. 9
   j. 10 - Extremely important
36. People vape for many reasons. Compared with all of your reasons for vaping, how important is vaping cannabis to control your weight? (if 1 is not at all and 10 is extremely important)

a. 1- Not at all
b. 2
c. 3
d. 4
e. 5
f. 6
g. 7
h. 8
i. 9
j. 10 - Extremely important

37. How much does cannabis vaping help you to control your weight? (if 1 is not at all and 10 is very much)
    a. 1- Not at all
    b. 2
    c. 3
    d. 4
    e. 5
    f. 6
    g. 7
    h. 8
    i. 9
    j. 10 – Very much
38. If you were thinking about making a quit attempt... Would you be concerned about gaining weight as a result of quitting?
    a. Yes
    b. No
39. If you were thinking about making a quit attempt... How concerned would you be about gaining weight as a result of quitting? (if 1 is not at all concerned and 10 is extremely concerned)
    a. 1- Not at all
    b. 2
    c. 3
    d. 4
    e. 5
    f. 6
    g. 7
    h. 8
    i. 9
    j. 10 – Extremely concerned
40. If you were thinking about making a quit attempt... How confident would you be that you could avoid gaining weight after quitting? (if 1 is not at all confident and 10 is extremely confident)
    a. 1- Not at all
    b. 2

c. 3
d. 4
e. 5
f. 6
g. 7
h. 8
i. 9
j. 10 – Extremely concerned

41. During any of your past quit attempts, did you use any of the following products: nicotine patch, nicotine gum/lozenge, nicotine nasal spray, nicotine inhaler, Chantix or Varenicline, Zyban, Bupropion or Wellbutrin or any other product?
    a. Yes
    b. No

Section VII: Penn State Electronic Cigarette Dependence Index (PSECDI)

1. How many TIMES per DAY do you use your electronic cigarette? (assume one 'TIME' consists of around 15 puffs, or lasts around 10 minutes)
2. On days that you can use your electronic cigarette freely, how soon after you wake up do you first use your electronic cigarette?
3. Do you sometimes awaken at night to use your electronic cigarette?
   - Yes
   - No
4. If yes, how many nights per week do you typically awaken to use your electronic cigarette?
5. Do you use your electronic cigarette now because it is really hard to quit using your electronic cigarette?
   - Yes
   - No
6. Do you ever have strong cravings to use an electronic cigarette?
   - Yes
   - No
7. Over the past week, how strong have the urges to use an electronic cigarette been?
   - No urges
   - Slight
   - Moderate
   - Strong
   - Very Strong
   - Extremely Strong
8. Is it hard to keep from using an electronic cigarette in places where you are not supposed to?
   - Yes
   - No

9. When you haven’t used an electronic cigarette for a while or when you tried to stop using did you feel more irritable because you couldn’t use an electronic cigarette?
   - Yes
   - No
10. When you haven’t used an electronic cigarette for a while or when you tried to stop using did you feel nervous, restless, or anxious because you couldn’t use an electronic cigarette?
   - Yes
   - No

Section VIII: Benefits, Harms, and Reasons for Use- Nicotine E-Cig

Instructions: Which of the following are the reasons why you use your electronic cigarette? (Check all that apply)

1. Using electronic cigarettes ...
   a. helps me cut down on the amount of cigarettes I smoke
   b. helped me quit smoking
   c. is good to use in places where smoking is not allowed
   d. makes it so I don't have to go outside to smoke
   e. is less harmful to my health than smoking cigarettes
   f. reduces harmful effects on my family or friends
   g. is cheaper than smoking cigarettes
   h. reduces the risk of lung cancer
   i. reduces the risk of mouth or throat cancer
   j. reduces my coughing with mucous
   k. makes me less short of breath, improves my breathing
   l. makes my hands and clothes smell less bad
   m. reduces my bad breath and bad odors
   n. improves my sense of smell and ability to taste
   o. tastes good
   p. feels good when inhaling
   q. reduces my urges or craving to smoke
   r. makes it easier to keep from smoking cigarettes
   s. keeps me from bothering other people with my smoke
   t. takes away my craving to smoke faster than cigarette smoking does
   u. gives me a much or more nicotine than I can get by smoking cigarettes
   v. reduces nicotine withdrawal
   w. helps me sleep better
   x. produces large clouds and shapes from vapor
   y. is trendy
   z. is more socially acceptable than smoking
   aa. allows me to experiment with flavors

bb. gets me high
cc. makes me more alert
dd. is healther than combustible cigarettes

Instructions: Which of the following concerns do you have about electronic cigarettes? (Check all that apply)

2. Using a electronic cigarettes ...
    a. The vapor they make contains other chemicals which are not safe; they hurt my health.
    b. I still get nicotine so I stay addicted.
    c. The nicotine vapor from electronic cigarettes causes lung cancer.
    d. The nicotine vapor from electronic cigarettes causes wet cough with mucous.
    e. The nicotine vapor from electronic cigarettes causes a dry cough.
    f. The nicotine vapor from electronic cigarettes causes lung problems.
    g. The nicotine vapor from electronic cigarettes causes mouth or throat cancer.
    h. The nicotine vapor from electronic cigarettes makes me short of breath.
    i. Using them burns my throat.
    j. Using them gives me a dry mouth or dry throat.
    k. Electronic cigarettes have toxic substances in them.
    l. Electronic cigarettes don't stop me from having urges or cravings to smoke.
    m. I don't get enough nicotine from electronic cigarettes.
    n. It's too difficult to adjust how much nicotine I get with electronic cigarettes.
    o. Electronic cigarettes are addicting.
    p. Electronic cigarettes don't help me quit smoking.
    q. If I use electronic cigarettes to quit smoking, I'll just go back to smoking when I stop using them.
    r. Using electronic cigarettes gives me headaches or nausea or makes me feel dizzy.
    s. Electronic cigarettes taste bad.
    t. Electronic cigarettes cause me to gain weight.
3. What are the primary reasons for using your vape or electronic cigarette?
    a. Ease of use
    b. Quitting a combustible product
    c. Health benefits
    d. Taste
    e. Feeling (high or alertness)
    f. Satisfaction
    g. Less obvious than smoking a combustible
    h. New and interesting
    i. Cheaper than other products

j. Dependence or addiction
k. To lose weight
l. Please tell us more about why you choose to use an e-cig.

Section IX: DFAQ-CU Inventory

1. Which of the following best captures when you last used cannabis?
    a. Over a year ago
    b. 9-12 months ago
    c. 6-9 months ago
    d. 3-6 months ago
    e. 1-3 months ago
    f. Less than 1 month ago
    g. Last week
    h. This week
    i. Yesterday
    j. Today
2. Which of the following best captures the average frequency you currently use cannabis?
    a. I do not use cannabis
    b. Less than once a year
    c. Once a year
    d. Once every 3-6 months (2-4 times/yr)
    e. Once every 2 months (6 times/yr)
    f. Once a month (12 times/yr)
    g. 2-3 times a month
    h. Once a week
    i. Twice a week
    j. 3-4 times a week
    k. 5-6 times a week
    l. Once a day
    m. More than once a day
3. Which of the following best captures how long you have been using cannabis at this frequency?
    a. Less than 1 month
    b. 1-3 months
    c. 3-6 months
    d. 6-9 months
    e. 9-12 months
    f. 1-2 years
    g. 2-3 years
    h. 3-5 years
    i. 5-10 years
    j. 10-15 years

    k. 15-20 years
    l. More than 20 years
4. Before the period of time you indicated above, how frequently did you use cannabis?
    a. I do not use cannabis
    b. Less than once a year
    c. Once a year
    d. Once every 3-6 months (2-4 times/yr)
    e. Once every 2 months (6 times/yr)
    f. Once a month (12 times/yr)
    g. 2-3 times a month
    h. Once a week
    i. Twice a week
    j. 3-4 times a week
    k. 5-6 times a week
    l. Once a day
    m. More than once a day
5. How many days of the past week did you use cannabis?
    a. 0 days
    b. 1 day
    c. 2 days
    d. 3 days
    e. 4 days
    f. 5 days
    g. 6 days
    h. 7 days
6. Approximately how many days of the past month did you use cannabis?
7. Which of the following best captures the number of times you have used cannabis in your entire life?
    a. 1-5 times in my life
    b. 6-10 times in my life
    c. 11-50 times in my life
    d. 51-100 times in my life
    e. 101-500 times in my life
    f. 501-1000 times in my life
    g. 1001-200 time in my life
    h. 2001-5000 times in my life
    i. 5001-10,000 times in my life
    j. More than 10,000 times in my life
8. Which of the following best captures your pattern of cannabis use throughout the week?
    a. I do not use cannabis at all
    b. I only use cannabis on weekends
    c. I only use cannabis on weekdays

    d. I use cannabis on weekends and weekdays
9. How many hours after waking up do you typically first use cannabis?
    a. I do not use cannabis
    b. 12-18 hours after waking up
    c. 9-12 hours after waking up
    d. 6-9 hours after waking up
    e. 3-6 hours after waking up
    f. 1-3 hours after waking up
    g. Within 1 hour of waking up
    h. Within 30 minutes of waking up
    i. Immediately upon waking up
10. How many times a day, on a typical weekday, do you use cannabis?
11. How many times a day, on a typical weekend, do you use cannabis?
12. What is the primary method you use to ingest cannabis?
    a. I do not use cannabis
    b. Joints
    c. Blunts (cigar sized joints)
    d. Hand pipe
    e. Bong (water pipe)
    f. Hookah
    g. Vaporizer (e.g. Volcano, Vape pen)
    h. Edibles
    i. Other
13. Which of the following other methods to ingest cannabis do you use regularly? (at least 25% of the time use your cannabis)? [Mark all that apply]
    a. None
    b. Joints
    c. Blunts (cigar sized joints)
    d. Hand pipe
    e. Bong (water pipe)
    f. Hookah
    g. Vaporizer (e.g. Volcano, Vape pen)
    h. Edibles
    i. Other
14. What is the primary form of cannabis you use?
    a. None
    b. Leaf Marijuana
    c. Concentrates (e.g. Oil, Wax, Shatter, Butane Hash Oil, Dabs)
    d. Edibles
    e. Other
15. Please use the image below to refer to various quantities of marijuana. The image is not to scale; the dollar bill is included to help provide size perspective. For questions 16 to 18 below, clearly indicate the number of grams of marijuana you use with a number between 0 to 100. Do NOT include other forms of cannabis

you may use (such as concentrates). You may use up to 3 decimals to indicate amounts under 1 gram. Note: 1/8 of a gram = 0.125 grams, quarter of a gram = 0.25 grams, half of a gram = 0.5 grams, three fourths of a gram = 0.75 grams. one eighth of a ounce = 3.5 grams, quarter of an ounce = 7 grams, half ounce = 14 grams, 1 ounce = 28 grams

16. In a typical session, how much marijuana do you personally use?
17. On a typical day you use marijuana, how much do you personally use?
18. In a typical week you use marijuana, how much marijuana do you personally use?
19. On a typical day you use marijuana, how many sessions do you have?
20. What is the average THC content of the marijuana you typically use? Leave blank if you do not know.
    a. 0-4%
    b. 5-9%
    c. 10-14%
    d. 15-19%
    e. 20-24%
    f. 25-30%
    g. Greater than 30%
21. In a typical session you use cannabis concentrates, how many hits do you personally take?
22. On a typical day you use cannabis concentrates, how many hits do you personally take?
23. How many hits of cannabis concentrates did you personally take yesterday?
24. On a typical day you use cannabis concentrates, how many sessions do you have?
25. What is the average THC content of the concentrates you typically use? Leave blank if you do not know.
    a. 0-9%
    b. 10-19%
    c. 20-29%
    d. 30-29%
    e. 40-49%
    f. 50-59%
    g. 60-69%
    h. 70-79%
    i. 80-90%
    j. Greater than 90%
26. When you eat edibles how many milligrams of THC do you personally ingest in a typical session?
27. How many years in total have you used cannabis?
28. How old were you when you FIRST tried cannabis?
29. Has there been any time in your life when you used cannabis regularly (2 or more times per month for 6 months or longer)?
    a. Yes
    b. No

30. How old were you when you FIRST STARTED using cannabis regularly (2 or more times/month)?
31. Has there been any time in your life when you used cannabis on a daily or near daily basis for 6 months or longer?
    a. Yes
    b. No
32. How old were you when you FIRST STARTED using cannabis on a daily or near daily basis?
33. Which of the following best captures the average frequency that you used cannabis before the age of 16?
    a. More than once a day
    b. Once a day
    c. 5-6 times a week
    d. 3-4 times a week
    e. Twice a week
    f. Once a week
    g. 2-3 times a month
    h. Once a month
    i. Once every 2 months (6 times/yr.)
    j. Once every 3-6 months (2-4 times/yr.)
    k. Once a year
    l. Less than once a year
    m. Never
34. Do you have a medical marijuana card registered with your state?
    a. Yes
    b. No
35. Do you have a physician's recommendation to use cannabis for medicinal purposes?
    a. No
    b. Yes
    c. Yes, but I use it for both medicinal and recreational purposes
36. Which medical condition(s) do you use cannabis for?
37. What percentage of the time do you use cannabis for recreational (rather than medicinal) purposes?

Section X: Cannabis Severity of Dependence Scale (SDS)

During the past year…

1. Did you think your use of cannabis was out of control?
    a. Never/almost never
    b. Sometimes
    c. Often
    d. Always/nearly always
2. Did the prospect of missing a dose of cannabis make you anxious or worried?

    a. Never/almost never
    b. Sometimes
    c. Often
    d. Always/nearly always
3. Did you worry about your use of cannabis?
    a. Never/almost never
    b. Sometimes
    c. Often
    d. Always/nearly always
4. Did you wish you could stop the use of cannabis?
    a. Never/almost never
    b. Sometimes
    c. Often
    d. Always/nearly always
5. How difficult did you find it to stop, or go without cannabis?
    a. Not Difficult
    b. Quite Difficult
    c. Very Difficult
    d. Impossible

Section XI: Cannabis Vape Use History

Instructions: The following questions are about your use of vaporized cannabis e-liquid. Cannabis e-liquid includes THC, CBD, or other products of leaf or synthetic cannabis or marijuana. For this questionnaire, we are NOT asking about other ways that you might use marijuana or cannabis (e.g., joints, bowls, edibles). We are also NOT asking about your use of vapes that contain other products (e.g., nicotine, flavorants only).

1. We refer to your cannabis vaping device as a cannabis vape in this survey. What do you call your device?
2. At what age did you first try a cannabis vape?
3. At what age did you first begin to use a cannabis vape regularly?
4. How long have you been using a cannabis vape? Days, months, or years?
    a. Days
    b. Months
    c. Years
5. How many days?
6. How many months?
7. How many years?
8. Were you using cannabis regularly before you began using your cannabis vape?
    a. Yes
    b. No
9. Which of the following did you use? (check all that apply)
    a. smoking (e.g. joints, blunts, bowls)

b. vaporizing leaf (e.g. volcano)
c. hookah or water bong
d. dabbing (concentrated oil or resin)
e. edibles
f. tincture
g. other

10. On how many days of the past 30 have you used your cannabis vape?
11. When you use your cannabis vape, how many puffs do you typically take in one use session?
12. When you use your cannabis vape, how many minutes do you typically use it for at one time?
13. How often do you "stealth vape" by using your cannabis vape discreetly in places or situations where it may not be allowed or socially acceptable?
    a. Never
    b. Rarely
    c. A few times a month
    d. A few times a week
    e. Daily
14. What type of device do you use most often with your cannabis e-liquid? Please select the type that most closely resembles your device.
    a. Type 1
    b. Type 2
    c. Type 3
    d. Type 4
15. Is your cannabis vape a one-time-use device? (i.e., you cannot replace the cartridge and the battery is not rechargeable)
    a. Yes
    b. No
16. What percentage of THC is in the cannabis liquid you use?
17. What percentage of CBD is in the cannabis liquid you use?
18. Please list any other ingredients that are in your cannabis e-liquid.
19. Where do you purchase the cannabis e-liquid that you typically use?
    a. Gas station
    b. Tobacco store or vape shop
    c. Online store
    d. Online market place (e.g. amazon, ebay)
    e. Friends/family
    f. Individual seller
    g. Dispensary
    h. Other
20. If you purchase your products somewhere other than listed above, please write in:
21. Do you typically use a cannabis e-liquid with flavor added?
    a. Yes

b. No

22. What flavor do you typically use?
23. Do you use more than one flavor?
    a. Yes
    b. No
24. What other flavors do you use?
25. Do you ever feel as though the e-liquids you purchase have been unsafe?
    a. Yes
    b. No
26. Do you have a method of verifying that the e-liquid you purchased is the product you intended to receive?
    a. Yes
    b. No
27. What is your primary method of verification?
    a. Holding e-liquid up to light
    b. Looking for bubble in e-liquid
    c. Looking at packaging for damage
    d. Smelling the e-liquid
    e. Checking the viscosity
    f. Other
28. Please tell us the other ways you verify the e-liquid.
29. Have you had any concerns for your health due to your cannabis vape use?
    a. Yes
    b. No
30. Have you previously used your cannabis vape for vaping liquids containing products other than cannabis? (e.g., nicotine)
    a. Yes
    b. No
31. What other liquids?
32. Do you use a cannabis vape to replace the use of leaf or other combustible forms of cannabis?
    a. Yes
    b. No
33. Have you EVER made a serious attempt to stop using your cannabis vape because you were TRYING to quit -- even if you stopped for less than a day?
    a. Yes
    b. No
34. How many times have you ever made a serious attempt to stop vaping cannabis because you were TRYING to quit -- even if you stopped for less than a day?
35. Of all the quit attempts you have made, how long did your longest quit attempt last? Was it days, months, or years?
    a. Days
    b. Months
    c. Years

36. How many days did your longest quit attempt last?
37. How many months did your longest quit attempt last?
38. How many years did your longest quit attempt last?
39. On your longest previous quit attempt, did you gain any weight?
    a. Yes
    b. No
40. How much weight did you gain on this quit attempt?
41. Did you lose this weight at some point when you resumed vaping cannabis?
    a. Yes
    b. No
42. How important is losing weight or maintaining your current weight compared with other personal health concerns? (if 1 is not at all and 10 is extremely important)
    a. 1-Not at all
    b. 2
    c. 3
    d. 4
    e. 5
    f. 6
    g. 7
    h. 8
    i. 9
    j. 10- Extremely important
43. People vape for many reasons. Compared with all of your reasons for vaping, how important is vaping cannabis to control your weight? (if 1 is not at all and 10 is extremely important)
    a. 1-Not at all
    b. 2
    c. 3
    d. 4
    e. 5
    f. 6
    g. 7
    h. 8
    i. 9
    j. 10- Extremely important
44. How much does cannabis vaping help you to control your weight? (if 1 is not at all and 10 is very much)
    a. 1-Not at all
    b. 2
    c. 3
    d. 4
    e. 5
    f. 6

g. 7
h. 8
i. 9
j. 10- Very Much

45. If you were thinking about making a quit attempt... Would you be concerned about gaining weight as a result of quitting?
    a. Yes
    b. No
46. If you were thinking about making a quit attempt... How concerned would you be about gaining weight as a result of quitting? (if 1 is not at all concerned and 10 is extremely concerned)
    a. 1-Not at all
    b. 2
    c. 3
    d. 4
    e. 5
    f. 6
    g. 7
    h. 8
    i. 9
    j. 10- Extremely concerned
47. If you were thinking about making a quit attempt... How confident would you be that you could avoid gaining weight after quitting? (if 1 is not at all confident and 10 is extremely confident)
    a. 1-Not at all
    b. 2
    c. 3
    d. 4
    e. 5
    f. 6
    g. 7
    h. 8
    i. 9
    j. 10- Extremely confident

Section XII: Penn State Cannabis Vape Dependence Index (PSCVDI)

Instructions: The following questions are about your use of vaporized cannabis e-liquid in the past 12 months. Cannabis e-liquid includes THC, CBD, or other products of leaf or synthetic cannabis or marijuana. For this questionnaire, we are NOT asking about other ways that you might use marijuana or cannabis (e.g., joints, bowls, edibles). We are also NOT asking about your use of vapes that contain other products (e.g., nicotine, flavorants only).

1. How many TIMES per DAY do you use your cannabis vape? (assume one 'TIME' consists of around 15 puffs, or lasts around 10 minutes)
2. On days that you can use your cannabis vape freely, how soon after you wake up do you first use your cannabis vape?
3. Do you sometimes awaken at night to use your cannabis vape?
   - Yes
   - No
4. If yes, how many nights per week do you typically awaken to use your cannabis vape?
5. Do you use your cannabis vape now because it is really hard to quit using your cannabis vape?
   - Yes
   - No
6. Do you ever have strong cravings to use your cannabis vape?
   - Yes
   - No
7. Over the past week, how strong have the urges to use a cannabis vape been?
   - No urges
   - Slight
   - Moderate
   - Strong
   - Very strong
   - Extremely strong
8. Is it hard to keep from using a cannabis vape in places where you are not supposed to?
   - Yes
   - No
9. When you haven't used a cannabis vape for a while or when you tried to stop using did you feel more irritable because you couldn't use a cannabis vape?
   - Yes
   - No
10. When you haven't used a cannabis vape for a while or when you tried to stop using did you feel nervous, restless or anxious because you couldn't use a cannabis vape?
    - Yes
    - No

Section XIII: DSM-V Cannabis Vape Use Disorder

1. During the times when you use your cannabis vape, do you end up using more of it then you planned when you started?
2. How often does this happen?
   a. Rarely
   b. A few times a month

    c. A few times a week
    d. Daily
3. Have you repeatedly wanted to reduce or control your use of your cannabis vape?
4. How often do you think about reducing or controlling your use?
    a. Rarely
    b. A few times a month
    c. A few times a week
    d. Daily
5. On the days that you use your cannabis vape, do you spend substantial time obtaining, using it, or recovering from its effects?
6. How much time on average?
    a. <1 hour
    b. 2-3 hours
    c. 4-7 hours
    d. 8 hours or more
7. Do you crave or have a strong desire or urge to use a cannabis vape?
8. On average, how strong is your desire or urge to use your cannabis vape most days?
    a. Not very strong
    b. Somewhat strong
    c. Strong
    d. Very strong
9. Do you spend less time meeting your responsibilities at work, at school, or at home because of your repeated use?
10. How often does this happen?
    a. Rarely
    b. A few times a month
    c. A few times a week
    d. Daily
11. If your cannabis vape use causes problems with your family or other people, do you still keep using it?
12. How often does your cannabis vape cause problems with your family or other people?
    a. Rarely
    b. A few times a month
    c. A few times a week
    d. Daily
13. Have you used your cannabis vape more than once in any situation where you or others were physically at risk, for example, driving a car, riding a motorbike, using machinery, boating, etc?
14. How often do you use your cannabis vape in these types of situations?
    a. Rarely
    b. A few times a month

    c. A few times a week
    d. Daily
15. Do you continue to use your cannabis vape, even though it is clear that is has caused or worsened psychological or physical problems?
16. In your opinion, how severe are the psychological or physical symptoms caused by your cannabis vape?
    a. Not very severe
    b. Somewhat severe
    c. Severe
    d. Very severe
17. Did you reduce or give up important work, social, or recreational activities because of your cannabis vape use?
18. How often do you reduce or give up activities because of your cannabis vape use?
    a. Rarely
    b. A few times a month
    c. A few times a week
    d. Daily
19. Do you need to use your cannabis vape a lot more in order to get the same effect that you got when you first started using it or do you get much less effect with continued use of the same amount?
20. How much more do you need to use to get the same effect?
    a. A little bit more
    b. Somewhat more
    c. A lot more
    d. I can no longer get the same effect as the first time I used it
21. When you cut down on heavy or prolonged use of your cannabis vape do you sometimes experience any of the following symptoms? (Check all that apply)
    a. Irritability, anger, or aggression
    b. Nervousness or anxiety
    c. Trouble sleeping
    d. Appetite or weight loss
    e. Restlessness
    f. Feeling depressed
    g. Significant discomfort from one of the following: stomach pain, tremors or shakes, sweating, hot flashes, chills, headaches
22. How severe are these symptoms for you typically when you stop using your cannabis vape for a while?
    a. Not very severe
    b. Somewhat severe
    c. Severe
    d. Very severe
23. Did you use your cannabis vape to reduce or avoid some of these symptoms?
24. How often do you use your cannabis vape to avoid some of these symptoms?

a. Rarely
b. A few times a month
c. A few times a week
d. Daily

25. How addicted are you to cannabis vaping? (0 - Not at all addicted to 100 - Extremely addicted)
    a. 0 - Not at all
    b. 50
    c. 100 - Extremely addicted

Section XIV: Perceived Benefits, Harms, and Reasons for Use – Cannabis Vape

Instructions: Which of the following are reasons why you use your THC cannabis vape? (Check all that apply). The term "smoking" refers to combustible marijuana in the form of joints, blunts, bowls, etc.

1. Using a cannabis e-cigarette/vape ...
    a. helps me cut down on the amount of marijuana I smoke
    b. helped me quit smoking marijuana
    c. is good to use in places where smoking marijuana is not allowed
    d. makes it so I don't have to go outside to smoke
    e. is less harmful to my health than smoking marijuana
    f. reduces harmful effects on my family or friends
    g. is cheaper than smoking marijuana
    h. reduces the risk of lung cancer
    i. reduces the risk of mouth or throat cancer
    j. reduces my coughing with mucous
    k. makes me less short of breath, improves my breathing
    l. makes my hands and clothes smell less bad
    m. reduces my bad breath and bad odors
    n. improves my sense of smell and ability to taste
    o. tastes good
    p. feels good when inhaling
    q. reduces my urges or craving to smoke
    r. makes it easier to keep from smoking marijuana
    s. keeps me from bothering other people with my smoke
    t. takes away my craving to smoke faster than marijuana smoking does
    u. gives me a bigger high than I can get by smoking marijuana
    v. reduces withdrawal
    w. helps me sleep better

Instructions: Think about reasons you would not use a cannabis e-cig/vape. Which of the following are reasons why you would not use them? Check all that apply..

2. Using a cannabis e-cigarette/vape ...
    a. The vapor they make contains other chemicals which are not safe; they hurt my health.
    b. I still get THC so I stay addicted.
    c. The vapor from electronic cigarettes causes lung cancer.
    d. The vapor from electronic cigarettes causes wet cough with mucous.
    e. The vapor from electronic cigarettes causes a dry cough.
    f. The vapor from electronic cigarettes causes lung problems.
    g. The vapor from electronic cigarettes causes mouth or throat cancer.
    h. The vapor from electronic cigarettes makes me short of breath.
    i. Using them burns my throat.
    j. Using them gives me a dry mouth or dry throat.
    k. Electronic cigarettes have toxic substances in them.
    l. Electronic cigarettes don't stop me from having urges or cravings to smoke.
    m. I don't get enough THC/cannabis from electronic cigarettes.
    n. It's too difficult to adjust how much THC/cannabis I get with electronic cigarettes.
    o. Electronic cigarettes are addicting.
    p. Electronic cigarettes don't help me quit smoking.
    q. If I use electronic cigarettes to quit smoking, I'll just go back to smoking when I stop using them.
    r. Using electronic cigarettes gives me headaches or nausea or makes me feel dizzy.
    s. Electronic cigarettes taste bad.
    t. Electronic cigarettes cause me to gain weight.

3. What are the primary reasons for using your cannabis vape?
    a. Ease of use
    b. Quitting a combustible product
    c. Health benefits
    d. Taste
    e. Feeling (high or alertness)
    f. Satisfaction
    g. Less obvious than smoking a combustible
    h. New and interesting
    i. Cheaper than other products
    j. Dependence or addiction
    k. To lose weight
    l. Please tell us more about why you choose to use a cannabis vape.

Section XV: Clinical COPD Questionnaire

Instructions: Please select the response that best describes how you have been feeling during the past week. (Only one response for each question).

On average, during the past week, how often did you feel:

1. Short of breath at rest?
    a. Never
    b. Hardly ever
    c. A few times
    d. Several times
    e. Many times
    f. A great many times
    g. Almost all the time
2. Short of breath doing physical activities?
    a. Never
    b. Hardly ever
    c. A few times
    d. Several times
    e. Many times
    f. A great many times
    g. Almost all the time
3. Concerned about getting a cold or your breathing getting worse?
    a. Never
    b. Hardly ever
    c. A few times
    d. Several times
    e. Many times
    f. A great many times
    g. Almost all the time
4. Depressed (down) because of your breathing problems?
    a. Never
    b. Hardly ever
    c. A few times
    d. Several times
    e. Many times
    f. A great many times
    g. Almost all the time

In general, during the past week, how much of the time:

5. Did you cough?
    a. Never
    b. Hardly ever
    c. A few times
    d. Several times
    e. Many times
    f. A great many times
    g. Almost all the time

6. Did you produce phlegm?
    a. Never
    b. Hardly ever
    c. A few times
    d. Several times
    e. Many times
    f. A great many times
    g. Almost all the time

On average, during the past week, how limited were you in these activities because of your breathing problems:

7. Strenuous physical activities (such as climbing stairs, hurrying, doing sports)?
    a. Not limited at all
    b. Very slightly limited
    c. Slightly limited
    d. Moderately limited
    e. Very limited
    f. Extremely limited
    g. Totally limited/or unable to do
8. Moderate physical activities (such as walking, housework, carrying things)?
    a. Not limited at all
    b. Very slightly limited
    c. Slightly limited
    d. Moderately limited
    e. Very limited
    f. Extremely limited
    g. Totally limited/or unable to do
9. Daily activities at home (such as dressing, washing yourself)?
    a. Not limited at all
    b. Very slightly limited
    c. Slightly limited
    d. Moderately limited
    e. Very limited
    f. Extremely limited
    g. Totally limited/or unable to do
10. Social activities (such as talking, being with children, visiting friends/relatives)?
    a. Not limited at all
    b. Very slightly limited
    c. Slightly limited
    d. Moderately limited
    e. Very limited
    f. Extremely limited
    g. Totally limited/or unable to do
11. *I have never brushed my teeth*

a. *Disagree*
b. *Slightly Disagree*
c. *Agree*
d. *Strongly Agree*

Section XVI: Patient Health Questionnaire 9 (PHQ9)

Instructions: Over the last 2 weeks how often have you been bothered by any of the following problems?

1. Little interest or pleasure in doing things
    a. 0- Not at all
    b. 1- Several days
    c. 2- More than half the days
    d. 3- Nearly every day
2. Feeling down, depressed, or hopeless
    a. 0- Not at all
    b. 1- Several days
    c. 2- More than half the days
    d. 3- Nearly every day
3. Trouble falling or staying asleep, or sleeping too much
    a. 0- Not at all
    b. 1- Several days
    c. 2- More than half the days
    d. 3- Nearly every day
4. Feeling tired or having little energy
    a. 0- Not at all
    b. 1- Several days
    c. 2- More than half the days
    d. 3- Nearly every day
5. Poor appetite or overeating
    a. 0- Not at all
    b. 1- Several days
    c. 2- More than half the days
    d. 3- Nearly every day
6. Feeling bad about yourself -- or that you are a failure or have let yourself or your family down
    a. 0- Not at all
    b. 1- Several days
    c. 2- More than half the days
    d. 3- Nearly every day
7. Trouble concentrating on things, such as reading the newspaper or watching television
    a. 0- Not at all

    b. 1- Several days
    c. 2- More than half the days
    d. 3- Nearly every day
8. Moving or speaking so slowly that other people could have noticed? Or the opposite -- being so fidgety or restless that you have been moving around a lot more than usual
    a. 0- Not at all
    b. 1- Several days
    c. 2- More than half the days
    d. 3- Nearly every day
9. Thoughts that you would be better off dead or of hurting yourself in some way
    a. 0- Not at all
    b. 1- Several days
    c. 2- More than half the days
    d. 3- Nearly every day
10. If you checked off any problems, how difficult have these problems made it for you to do your work, take care of things at home, or get along with other people?
    a. 1- Not difficult at all
    b. 2- Somewhat difficult
    c. 3- Very difficult
    d. 4- Extremely difficult

Section XVII: Generalized Anxiety Disorder 7 Item (GAD 7) Scale

Instructions: Over the last 2 weeks, how often have you been bothered by the following problems?

1. Feeling nervous, anxious, or on edge
    a. 0- Not at all
    b. 1- Several days
    c. 2- More than half the days
    d. 3- Nearly every day
2. Not being able to stop or control worrying
    a. 0- Not at all
    b. 1- Several days
    c. 2- More than half the days
    d. 3- Nearly every day
3. Worrying too much about different things
    a. 0- Not at all
    b. 1- Several days
    c. 2- More than half the days
    d. 3- Nearly every day
4. Trouble relaxing
    a. 0- Not at all
    b. 1- Several days
    c. 2- More than half the days

    d. 3- Nearly every day
5. Being so restless that it's hard to sit still
    a. 0- Not at all
    b. 1- Several days
    c. 2- More than half the days
    d. 3- Nearly every day
6. Becoming easily annoyed or irritable
    a. 0- Not at all
    b. 1- Several days
    c. 2- More than half the days
    d. 3- Nearly every day
7. Feeling afraid as if something awful might happen
    a. 0- Not at all
    b. 1- Several days
    c. 2- More than half the days
    d. 3- Nearly every day
8. If you checked off any problems, how difficult have these made it for you to do your work, take care of things at home, or get along with other people?
    a. 1- Not difficult at all
    b. 2- Somewhat difficult
    c. 3- Very difficult
    d. 4- Extremely difficult
9. *I have been to every country in the world*
    a. *Disagree*
    b. *Slightly Disagree*
    c. *Agree*
    d. *Strongly Agree*

Section XVIII: Perceived Stress Scale (PSS-10)

1. In the last month, how often have you been upset because of something that happened unexpectedly?
    a. 0 = Never
    b. 1 = Almost Never
    c. 2 = Sometimes
    d. 3 = Fairly Often
    e. 4 = Very Often
2. In the last month, how often have you felt that you were unable to control the important things in your life?
    a. 0 = Never
    b. 1 = Almost Never
    c. 2 = Sometimes
    d. 3 = Fairly Often
    e. 4 = Very Often
3. In the last month, how often have you felt nervous and "stressed"?

   a. 0 = Never
   b. 1 = Almost Never
   c. 2 = Sometimes
   d. 3 = Fairly Often
   e. 4 = Very Often
4. In the last month, how often have you felt confident about your ability to handle your personal problems?
   a. 0 = Never
   b. 1 = Almost Never
   c. 2 = Sometimes
   d. 3 = Fairly Often
   e. 4 = Very Often
5. In the last month, how often have you felt that things were going your way?
   a. 0 = Never
   b. 1 = Almost Never
   c. 2 = Sometimes
   d. 3 = Fairly Often
   e. 4 = Very Often
6. In the last month, how often have you found that you could not cope with all the things that you had to do?
   a. 0 = Never
   b. 1 = Almost Never
   c. 2 = Sometimes
   d. 3 = Fairly Often
   e. 4 = Very Often
7. In the last month, how often have you been able to control irritations in your life?
   a. 0 = Never
   b. 1 = Almost Never
   c. 2 = Sometimes
   d. 3 = Fairly Often
   e. 4 = Very Often
8. In the last month, how often have you felt that you were on top of things?
   a. 0 = Never
   b. 1 = Almost Never
   c. 2 = Sometimes
   d. 3 = Fairly Often
   e. 4 = Very Often
9. In the last month, how often have you been angered because of things that were outside of your control?
   a. 0 = Never
   b. 1 = Almost Never
   c. 2 = Sometimes
   d. 3 = Fairly Often
   e. 4 = Very Often

10. In the last month, how often have you felt difficulties were piling up so high that you could not overcome them?
    a. 0 = Never
    b. 1 = Almost Never
    c. 2 = Sometimes
    d. 3 = Fairly Often
    e. 4 = Very Often
11. *Type "I am paying attention" in the text box*

Section XIX: Multidimensional Scale of Perceived Social Support (MSPSS)

Instructions: We are interested in how you feel about the following statements. Read each statement carefully. Indicate how you feel about each statement.

1. There is a special person who is around when I am in need
    a. Very strongly disagree
    b. Strongly disagree
    c. Mildly disagree
    d. Neutral
    e. Mildly agree
    f. Strongly agree
    g. Very strongly agree
2. There is a special person with whom I can share my joys and sorrows
    a. Very strongly disagree
    b. Strongly disagree
    c. Mildly disagree
    d. Neutral
    e. Mildly agree
    f. Strongly agree
    g. Very strongly agree
3. My family really tries to help me.
    a. Very strongly disagree
    b. Strongly disagree
    c. Mildly disagree
    d. Neutral
    e. Mildly agree
    f. Strongly agree
    g. Very strongly agree
4. I get the emotional help and support I need from my family
    a. Very strongly disagree
    b. Strongly disagree
    c. Mildly disagree
    d. Neutral
    e. Mildly agree

    f. Strongly agree
    g. Very strongly agree
5. I have a special person who is a real source of comfort to me
    a. Very strongly disagree
    b. Strongly disagree
    c. Mildly disagree
    d. Neutral
    e. Mildly agree
    f. Strongly agree
    g. Very strongly agree
6. My friends really try to help me.
    a. Very strongly disagree
    b. Strongly disagree
    c. Mildly disagree
    d. Neutral
    e. Mildly agree
    f. Strongly agree
    g. Very strongly agree
7. I can count on my friends when things go wrong
    a. Very strongly disagree
    b. Strongly disagree
    c. Mildly disagree
    d. Neutral
    e. Mildly agree
    f. Strongly agree
    g. Very strongly agree
8. I can talk about my problems with my family
    a. Very strongly disagree
    b. Strongly disagree
    c. Mildly disagree
    d. Neutral
    e. Mildly agree
    f. Strongly agree
    g. Very strongly agree
9. I have friends with whom I can share my joys and sorrows
    a. Very strongly disagree
    b. Strongly disagree
    c. Mildly disagree
    d. Neutral
    e. Mildly agree
    f. Strongly agree
    g. Very strongly agree
10. There is a special person in my life who cares about my feelings
    a. Very strongly disagree

    b. Strongly disagree
    c. Mildly disagree
    d. Neutral
    e. Mildly agree
    f. Strongly agree
    g. Very strongly agree
11. My family is willing to help me make decisions
    a. Very strongly disagree
    b. Strongly disagree
    c. Mildly disagree
    d. Neutral
    e. Mildly agree
    f. Strongly agree
    g. Very strongly agree
12. I can talk about my problems with my friends
    a. Very strongly disagree
    b. Strongly disagree
    c. Mildly disagree
    d. Neutral
    e. Mildly agree
    f. Strongly agree
    g. Very strongly agree

Section XX: Attention check:

1. *I have carefully read every survey item.*
    a. *Agree*
    b. *Disagree*
2. *I could have paid closer attention to the items than I did.*
    a. *Agree*
    b. *Disagree*
